# Supplementary material for: Design, Synthesis and Biological Evaluation of Neocryptolepine Derivatives as Potential Anti-Gastric Cancer Agents
Source: Int J Mol Sci. 2022 Oct 7;23(19):11924. doi: 10.3390/ijms231911924 (PMC9569688; doi:10.3390/ijms231911924)
Supplement: Supplementary file 1 [file ijms-23-11924-s001.zip › ijms-1949957-supplementary.pdf]

Supporting Information

Design, Synthesis and Biological Evaluation of Neocryptolepine Derivatives as Potential Anti-gastric Cancer Agents

Yunhao Ma [1,†], Yanan Tian [1,†], Zhongkun Zhou [1], Shude Chen [2], Kangjia Du [1], Hao Zhang [1], Xinrong Jiang [1], Juan Lu [1], Yuqing Niu [1], Lixue Tu [1], Jie Wang [1], Huanxiang Liu [1], Hongmei Zhu [1], Peng Chen [1,*], Yingqian Liu [1,*]

[1] School of Pharmacy, Lanzhou University, Lanzhou 730000, China

[2] The Second Clinical Medical College, Lanzhou University, Lanzhou 730000, China

[*] Correspondence: chenpeng@lzu.edu.cn (P.C.); yqliu@lzu.edu.cn (Y.L.); Tel./Fax: +86-931-8915686 (P.C. & Y.L.)

[†] These authors contributed equally to this work.

Table and Figure Caption

Table S1. The molecular docking scoring of compound **C5** and proteins

Table S2. The molecular docking scoring of compound **C8** and proteins

Figure S1. ^{1}H NMR spectra of compound **C5**

Figure S2. ^{13}C NMR spectra of compound **C5**

Figure S3. Mass spectrometry of compound **C5**

Figure S4. ^{1}H NMR spectra of compound **C8**

Figure S5. ^{13}C NMR spectra of compound **C8**

Figure S6. Mass spectrometry of compound **C8**

Figure S7. Purity test of compound **A1** by HPLC

Figure S8. Purity test of compound **A2** by HPLC

Figure S9. Purity test of compound **A3** by HPLC

Figure S10. Purity test of compound **A4** by HPLC

Figure S11. Purity test of compound **A5** by HPLC

Figure S12. Purity test of compound **A6** by HPLC

Figure S13. Purity test of compound **A7** by HPLC

Figure S14. Purity test of compound **A8** by HPLC

Figure S15. Purity test of compound **A9** by HPLC

Figure S16. Purity test of compound **A10** by HPLC

Figure S17. Purity test of compound **B1** by HPLC

Figure S18. Purity test of compound **B2** by HPLC

Figure S19. Purity test of compound **B3** by HPLC

Figure S20. Purity test of compound **B4** by HPLC

Figure S21. Purity test of compound **B5** by HPLC

Figure S22. Purity test of compound **B6** by HPLC

Figure S23. Purity test of compound **B7** by HPLC

Figure S24. Purity test of compound **B8** by HPLC

Figure S25. Purity test of compound **B9** by HPLC

Figure S26. Purity test of compound **C1** by HPLC

Figure S27. Purity test of compound **C2** by HPLC

Figure S28. Purity test of compound **C3** by HPLC

Figure S29. Purity test of compound **C4** by HPLC

Figure S30. Purity test of compound **C5** by HPLC

Figure S31. Purity test of compound **C6** by HPLC

Figure S32. Purity test of compound **C7** by HPLC

Figure S33. Purity test of compound **C8** by HPLC

Figure S34. Purity test of compound **C9** by HPLC

Figure S35. Purity test of compound **C10** by HPLC

Figure S36. Purity test of compound **D1** by HPLC

Figure S37. Purity test of compound **D2** by HPLC

Figure S38. Purity test of compound **D3** by HPLC

Figure S39. Purity test of compound **D4** by HPLC

Figure S40. Purity test of compound **D5** by HPLC

Figure S41. Purity test of compound **D6** by HPLC

Figure S42. Purity test of compound **E1** by HPLC

Table S1 The molecular docking scoring of compound **C5** and proteins

Number	Target	PDB ID	Resolution	Organisms	Docking score
1	AKT	6hhf	2.90	*Homo sapiens*	-8.529
2	Cyclin E1	5l2w	2.80	*Homo sapiens*	-8.171
3	PI3K	6gvf	2.50	*Homo sapiens*	-8.077
4	CDK2	5nev	2.97	*Homo sapiens*	-7.744
5	Cyclin A	6gue	1.99	*Homo sapiens*	-7.535
6	CDK6	3nux	2.70	*Homo sapiens*	-7.497
7	BCl-xl	7lh7	1.41	*Homo sapiens*	-7.377
8	MEK	5eym	2.70	*Homo sapiens*	-7.357
9	Raf	5fd2	2.89	*Homo sapiens*	-7.322
10	JNK	4y5h	2.06	*Homo sapiens*	-7.254
11	JAK	5tq6	2.06	*Homo sapiens*	-7.129
12	PARP	6bhv	2.30	*Homo sapiens*	-7.011
13	mTOR	4jt6	3.60	*Homo sapiens*	-6.81
14	GSK-3β	1pyx	2.40	*Homo sapiens*	-6.392
15	Cyclin D1	5vzu	2.70	*Homo sapiens*	-6.138
16	CDK7	1ua2	3.02	*Homo sapiens*	-5.656
17	Bax	4zie	1.80	*Homo sapiens*	-5.403
18	MAPK	6tca	3.70	*Homo sapiens*	-5.279
19	Bad	4ieh	2.10	*Homo sapiens*	-5.101
20	Bcl-2	6qgg	1.50	*Homo sapiens*	-5.035
21	Caspase-3	3kjf	2.00	*Homo sapiens*	-4.634
22	Ras	7jhp	2.77	*Homo sapiens*	-4.323
23	Casepase-9	1nw9	2.40	*Homo sapiens*	-4.076
24	STAT3	6nuq	3.15	*Homo sapiens*	-3.788
25	Wnt	6gbi	1.25	*Homo sapiens*	-2.6

Table S2 The molecular docking scoring of compound **C8** and proteins

Number	Target	PDB ID	Resolution	Organisms	Docking score
1	AKT	6hhf	2.90	*Homo sapiens*	-8.359
2	Cyclin E1	5l2w	2.80	*Homo sapiens*	-8.033
3	Cyclin A	6gue	1.99	*Homo sapiens*	-7.643
4	CDK6	3nux	2.70	*Homo sapiens*	-7.536
5	MEK	5eym	2.70	*Homo sapiens*	-7.518
6	Raf	5fd2	2.89	*Homo sapiens*	-7.494
7	JNK	4y5h	2.06	*Homo sapiens*	-7.316
8	CDK2	5nev	2.97	*Homo sapiens*	-7.256
9	BCl-xl	7lh7	1.41	*Homo sapiens*	-7.255
10	PARP	6bhv	2.30	*Homo sapiens*	-7.239
11	PI3K	6gvf	2.50	*Homo sapiens*	-7.123
12	mTOR	4jt6	3.60	*Homo sapiens*	-6.931
13	JAK	5tq6	2.06	*Homo sapiens*	-6.84
14	Cyclin D1	5vzu	2.70	*Homo sapiens*	-6.639
15	GSK-3β	1pyx	2.40	*Homo sapiens*	-6.185
16	Caspase-3	3kjf	2.00	*Homo sapiens*	-5.887
17	CDK7	1ua2	3.02	*Homo sapiens*	-5.501
18	Bad	4ieh	2.10	*Homo sapiens*	-5.211
19	MAPK	6tca	3.70	*Homo sapiens*	-5.107
20	Bcl-2	6qgg	1.50	*Homo sapiens*	-4.911
21	Ras	7jhp	2.77	*Homo sapiens*	-4.281
22	Casepase-9	1nw9	2.40	*Homo sapiens*	-3.995
23	NF-κB	5e6a	2.20	*Homo sapiens*	-3.878
24	STAT3	6nuq	3.15	*Homo sapiens*	-3.648
25	ERK	6hkn	2.33	*Homo sapiens*	-3.131

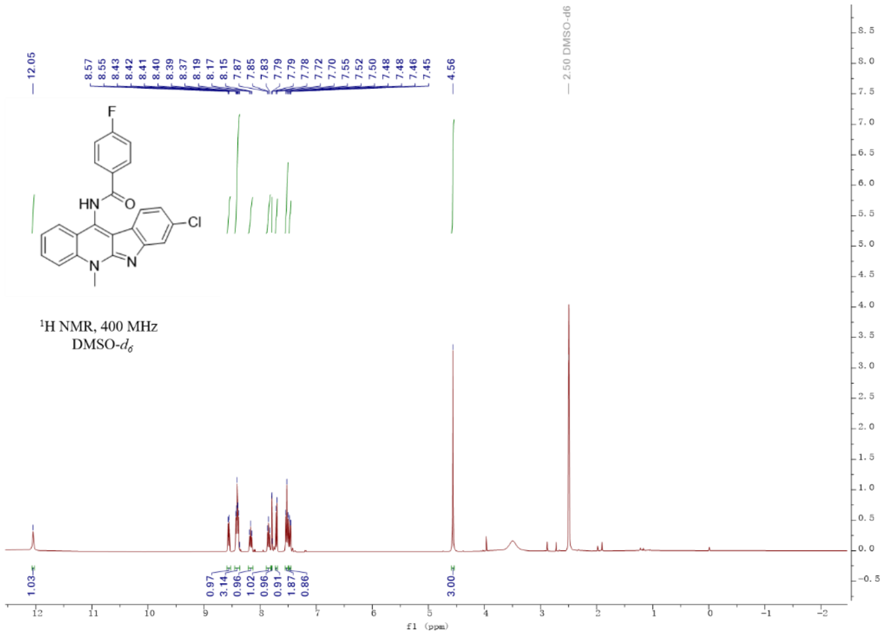

Figure S1. ^{1}H NMR spectra of compound **C5**

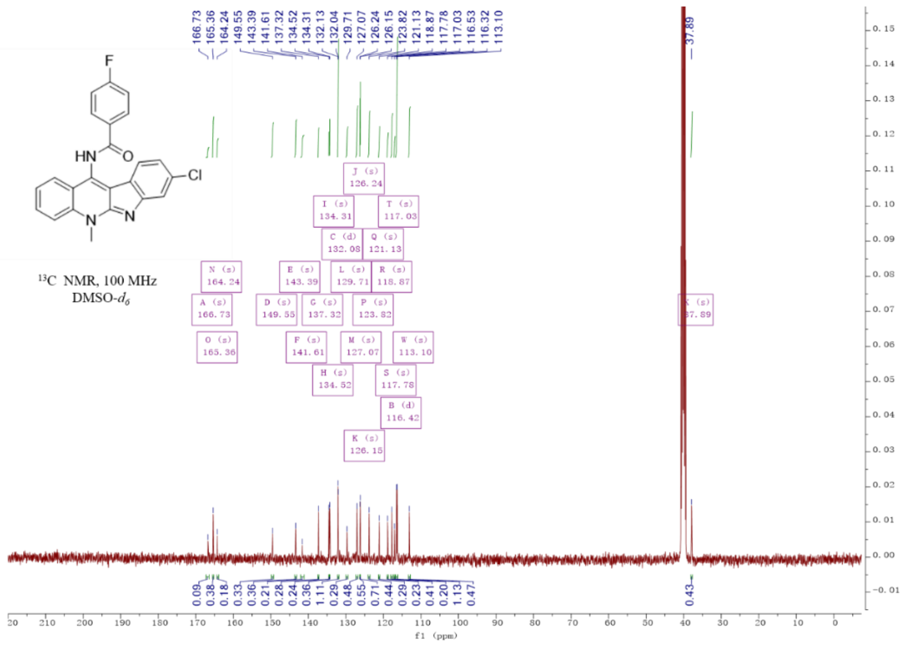

Figure S2. ^{13}C NMR spectra of compound **C5**

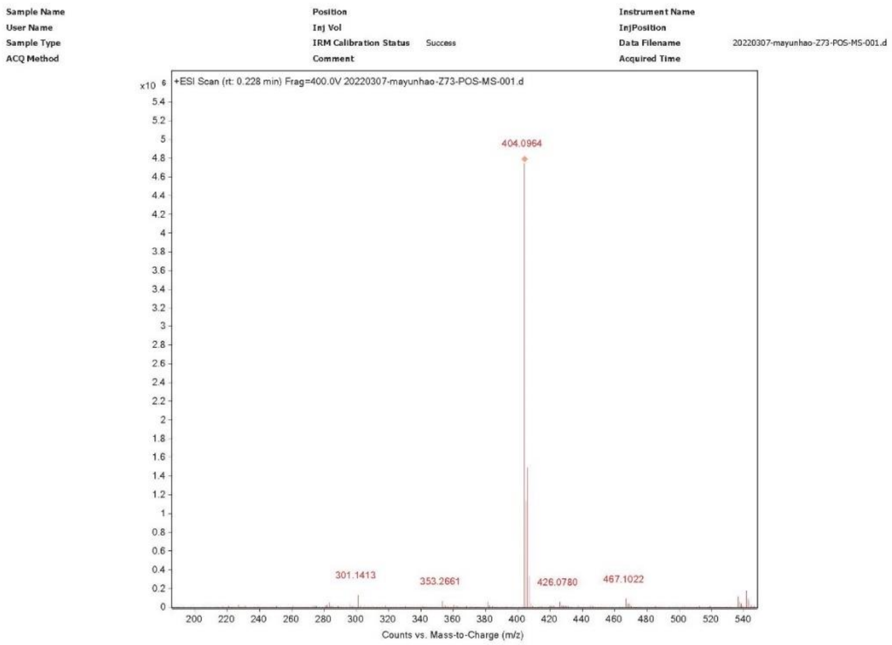

Figure S3. Mass spectrometry of compound **C5**

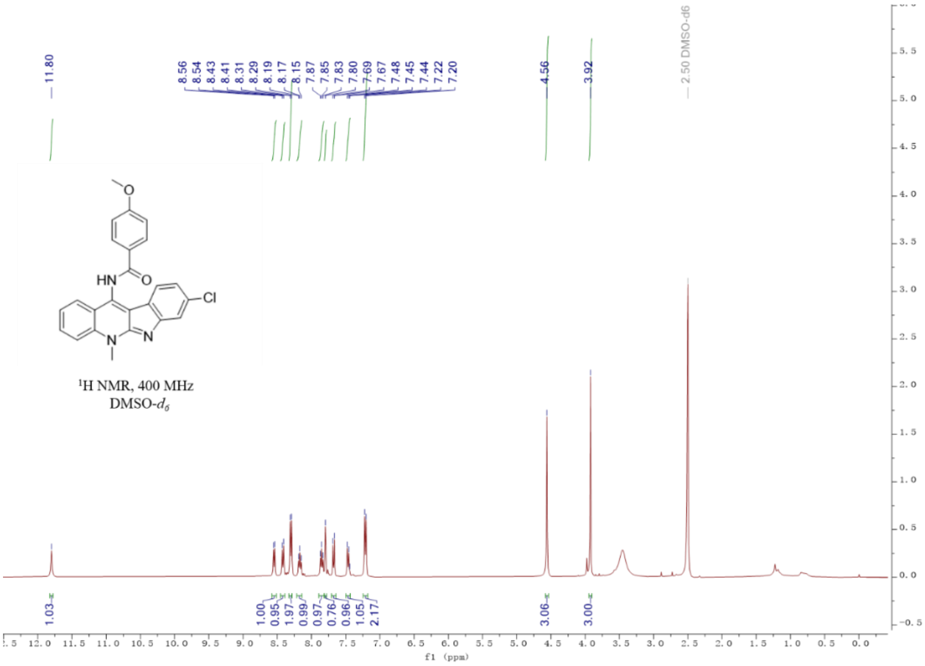

Figure S4. ^{1}H NMR spectra of compound **C8**

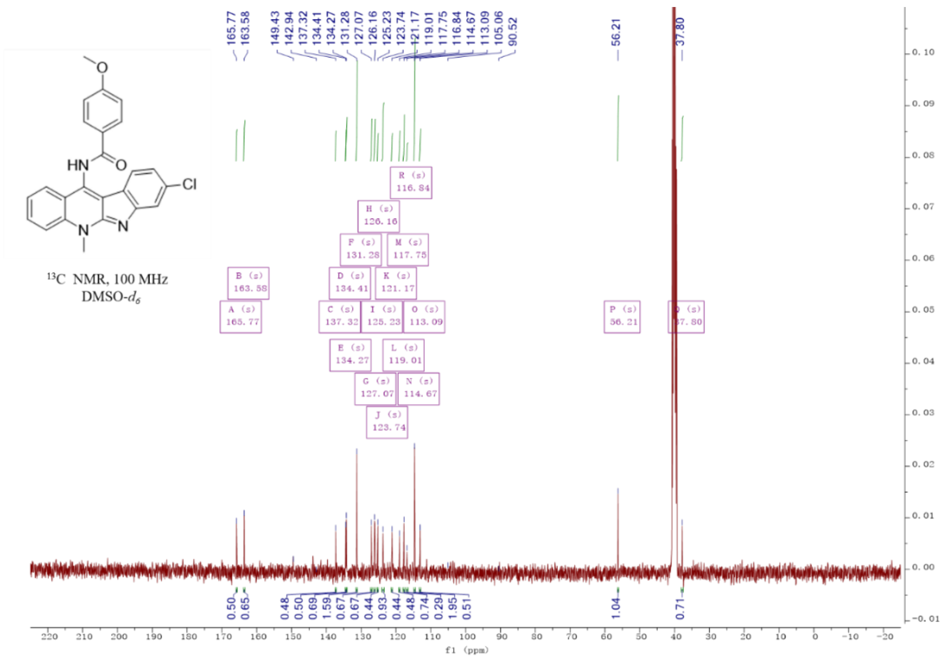

Figure S5. ^{13}C NMR spectra of compound **C8**

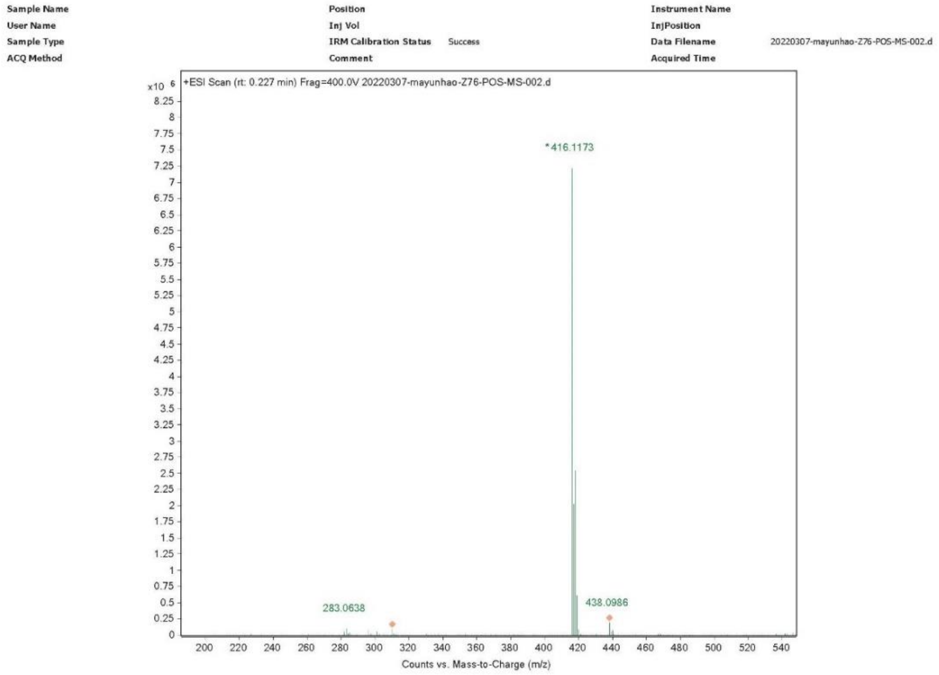

Figure S6. Mass spectrometry of compound **C8**

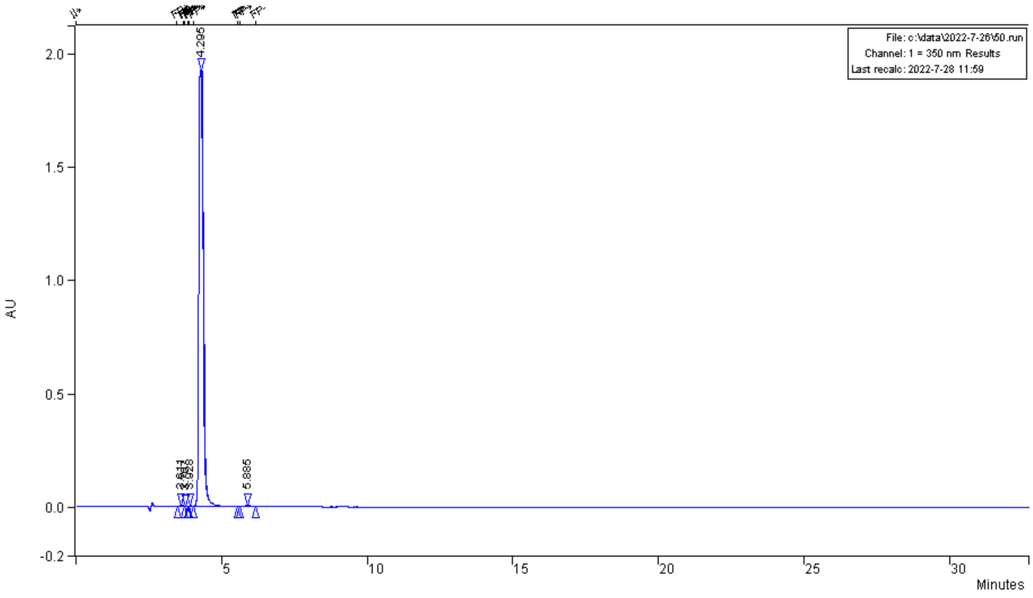

Figure S7. Purity test of compound **A1** by HPLC

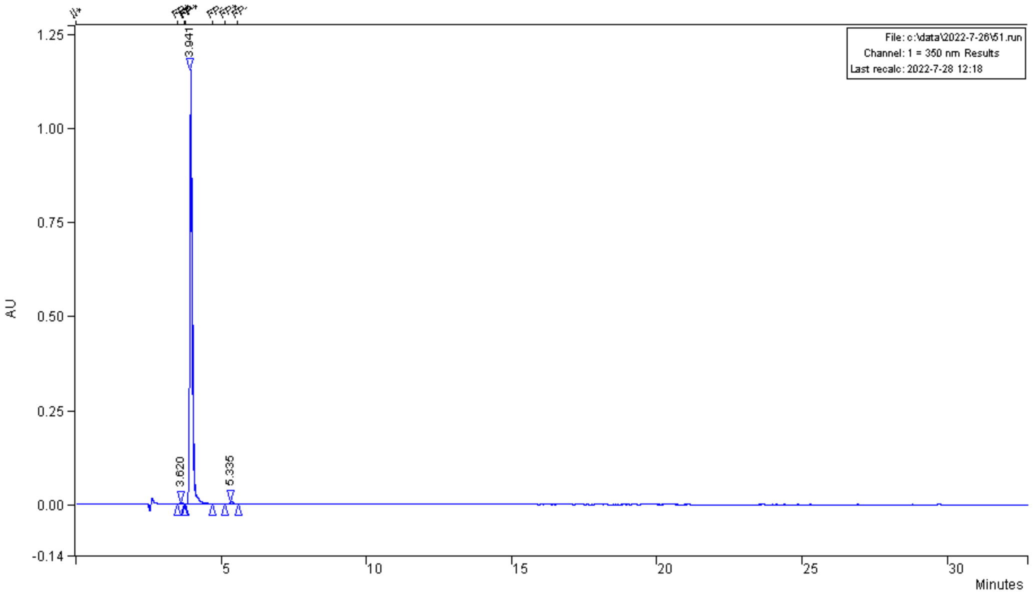

Figure S8. Purity test of compound **A2** by HPLC

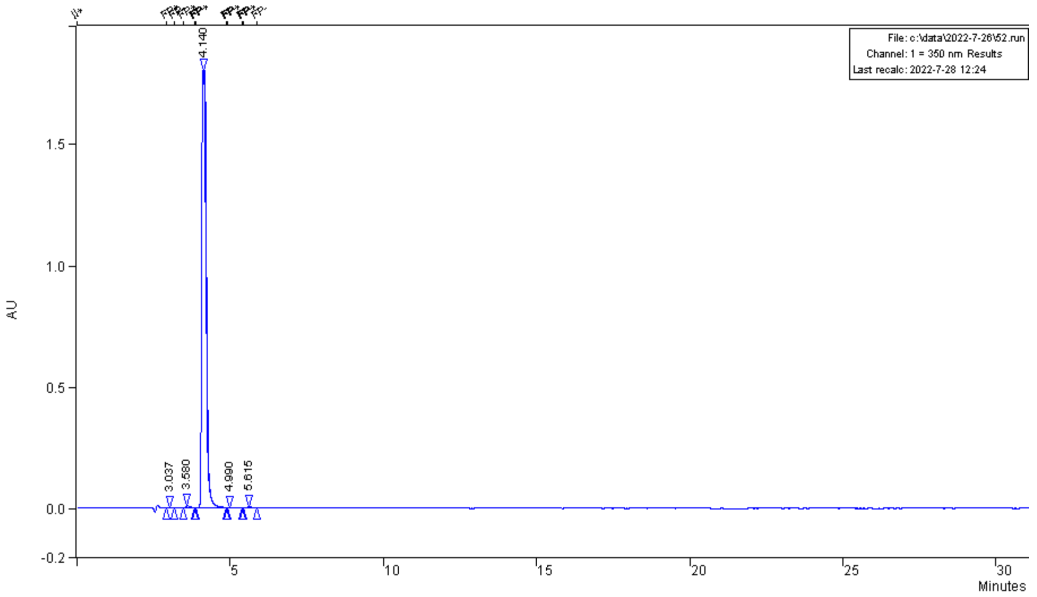

Figure S9. Purity test of compound **A3** by HPLC

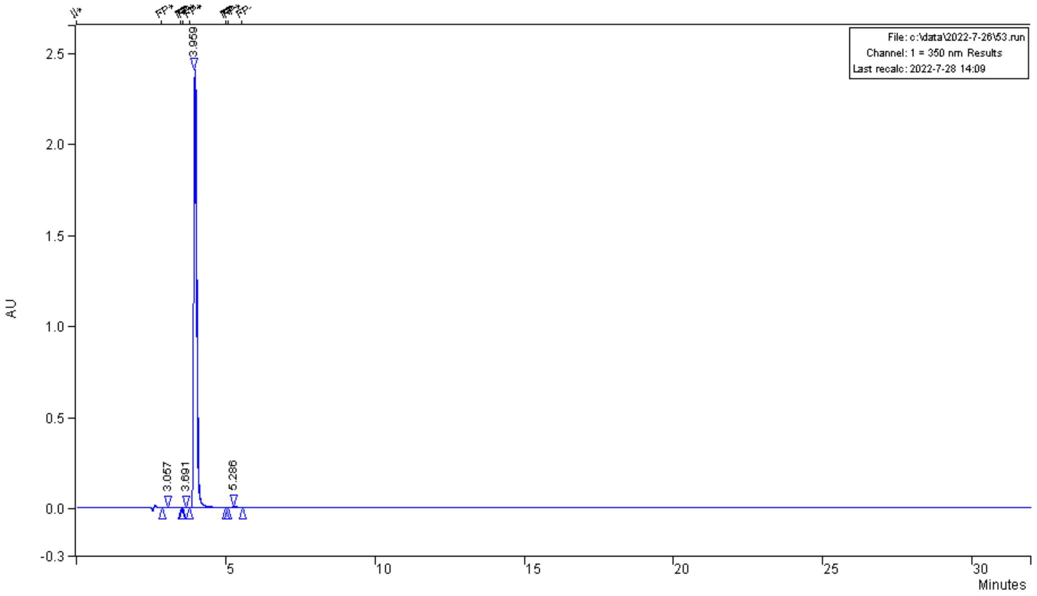

Figure S10. Purity test of compound **A4** by HPLC

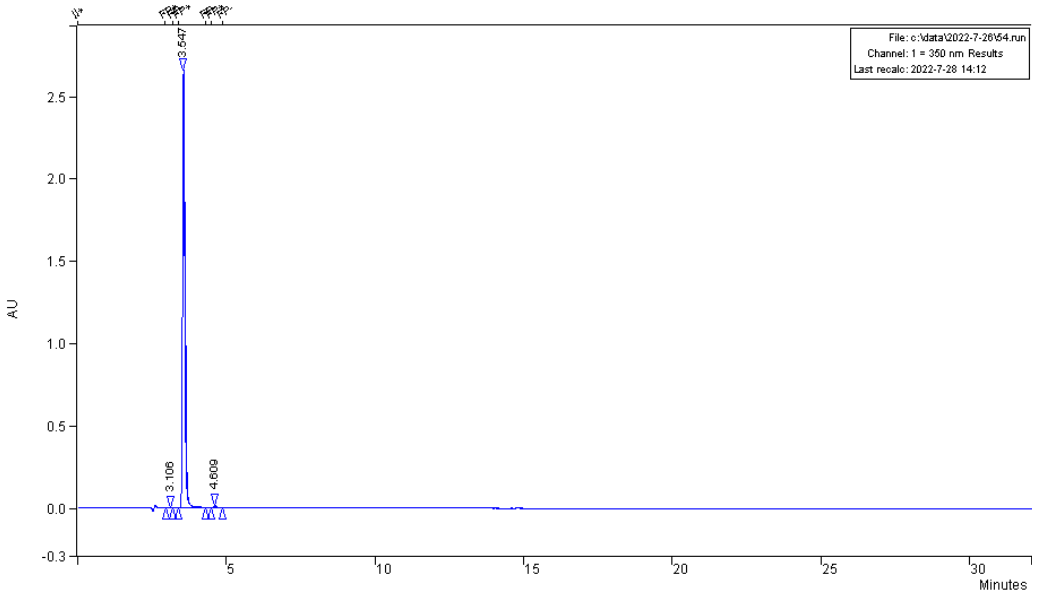

Figure S11. Purity test of compound **A5** by HPLC

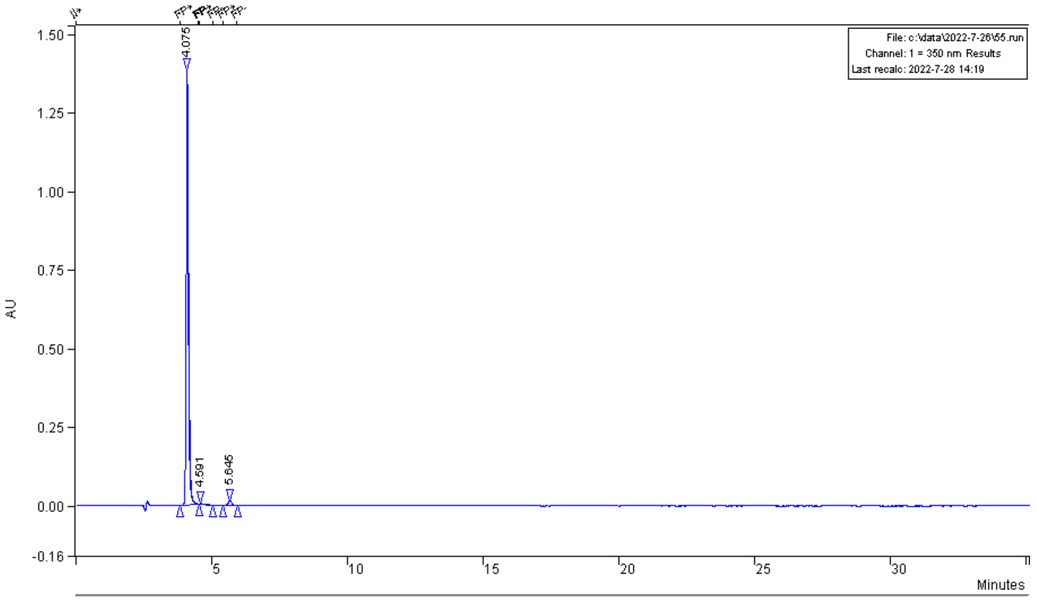

Figure S12. Purity test of compound **A6** by HPLC

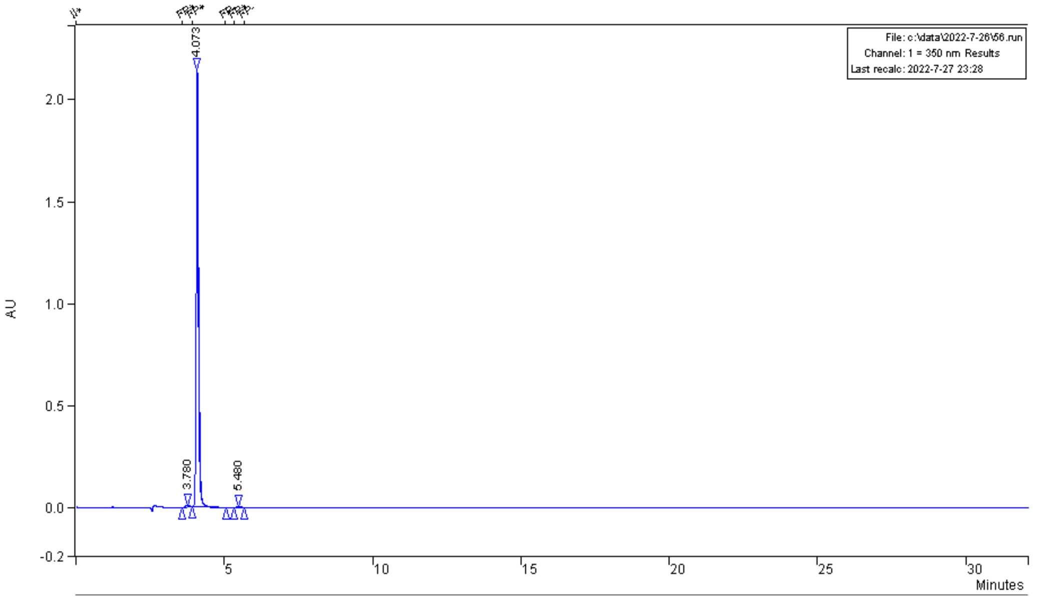

Figure S13. Purity test of compound **A7** by HPLC

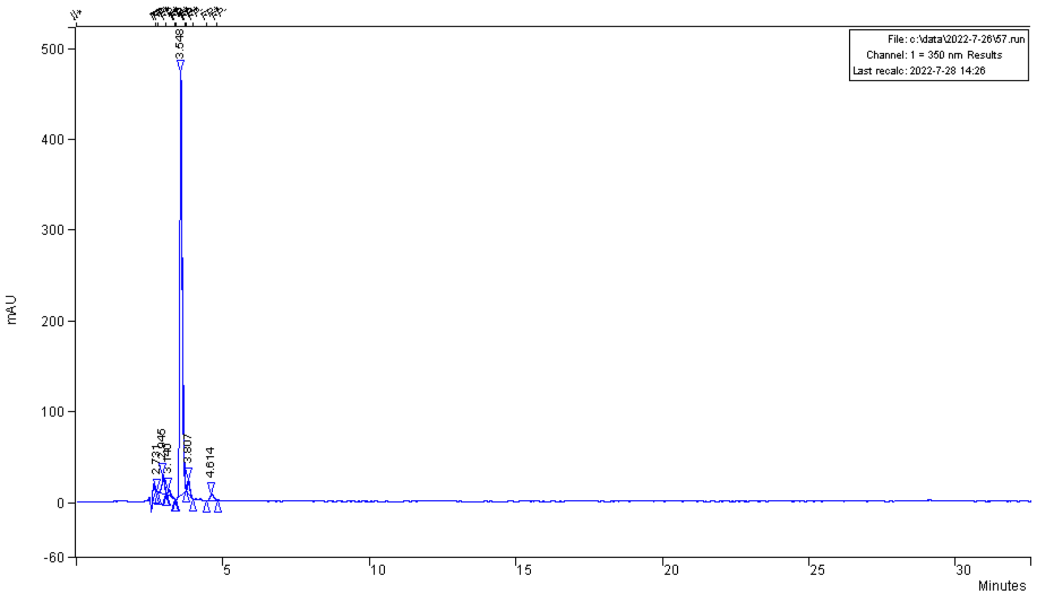

Figure S14. Purity test of compound **A8** by HPLC

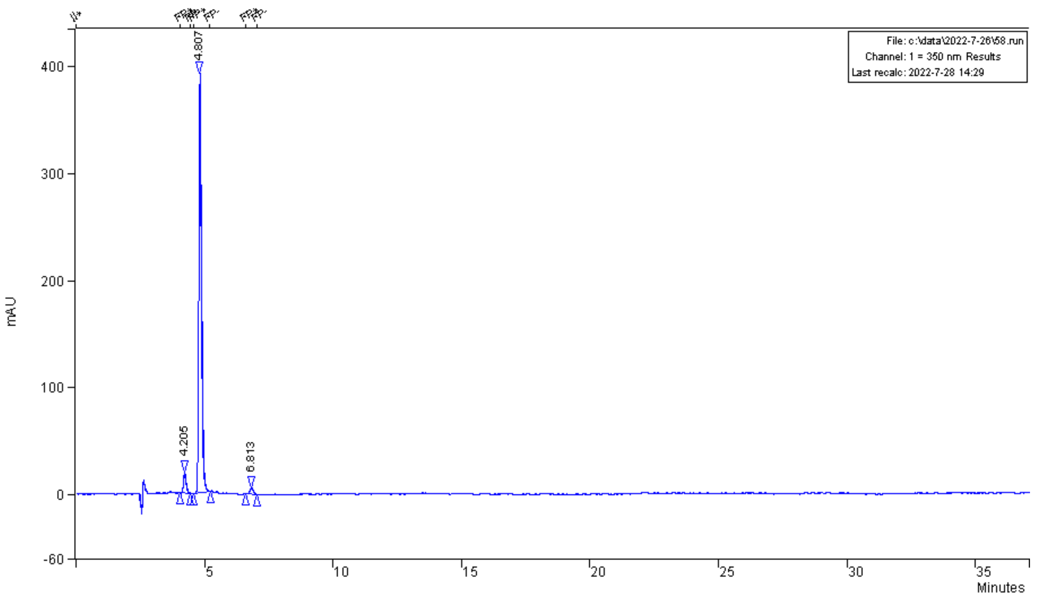

Figure S15. Purity test of compound **A9** by HPLC

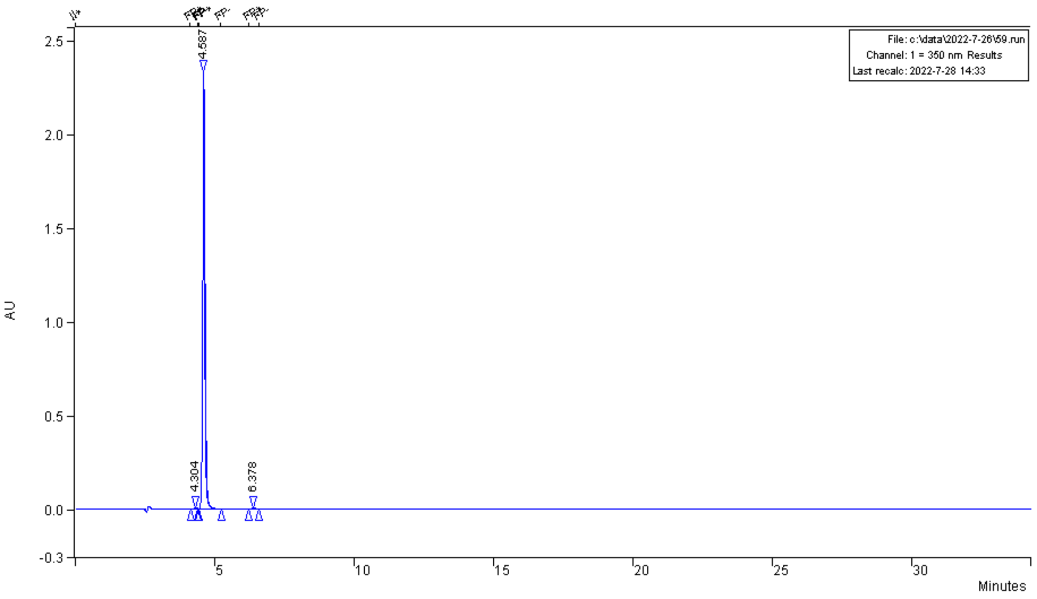

Figure S16. Purity test of compound **A10** by HPLC

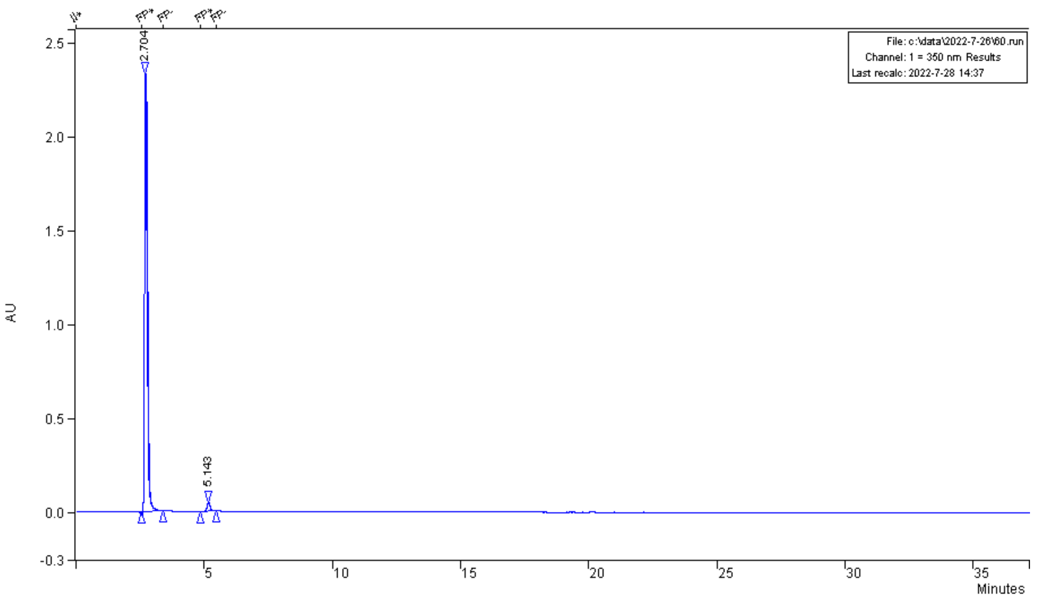

Figure S17. Purity test of compound **B1** by HPLC

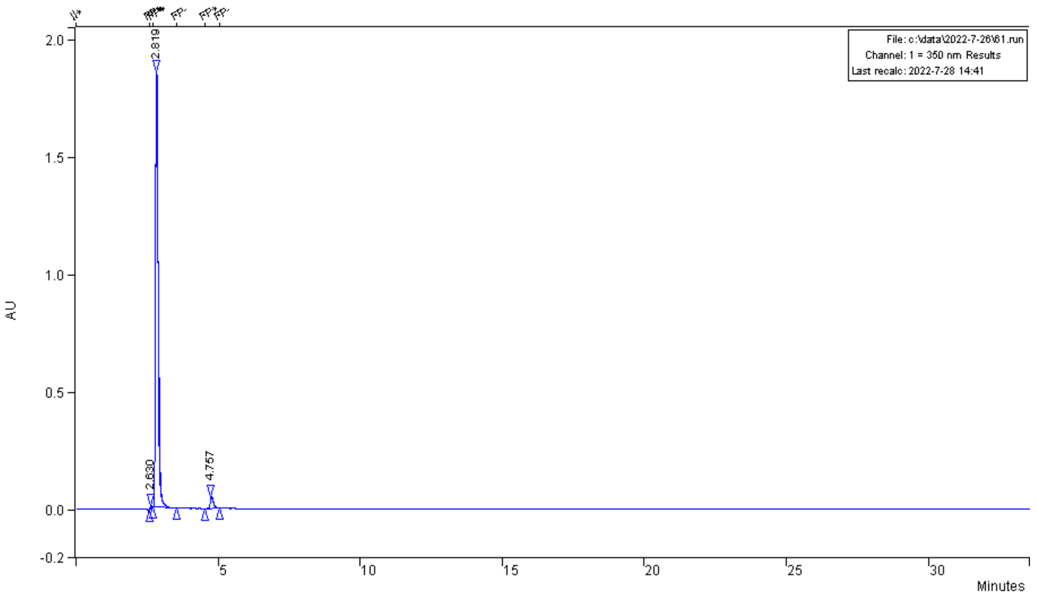

Figure S18. Purity test of compound **B2** by HPLC

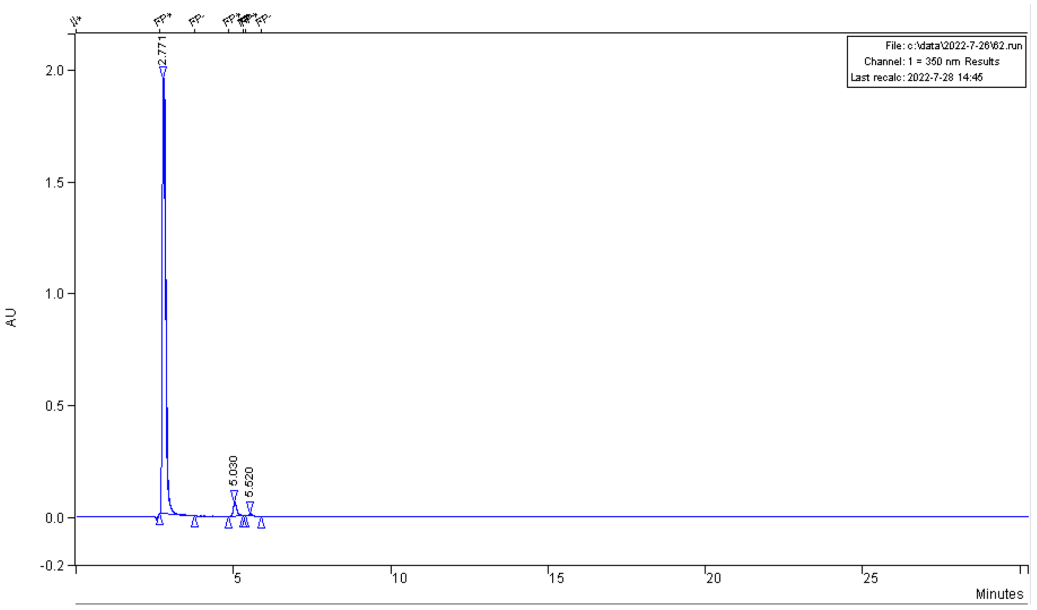

Figure S19. Purity test of compound **B3** by HPLC

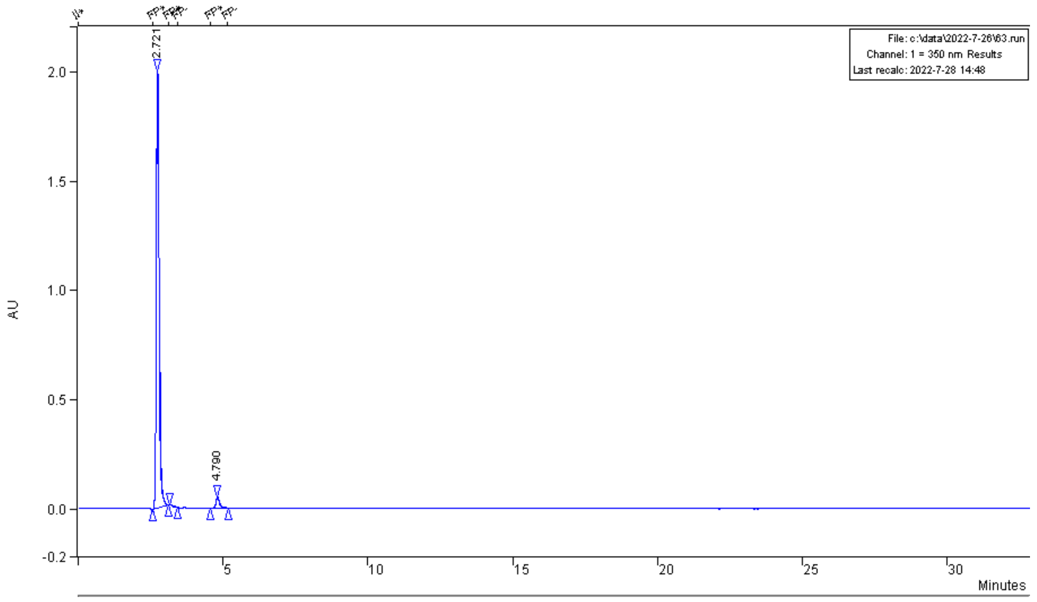

Figure S20. Purity test of compound **B4** by HPLC

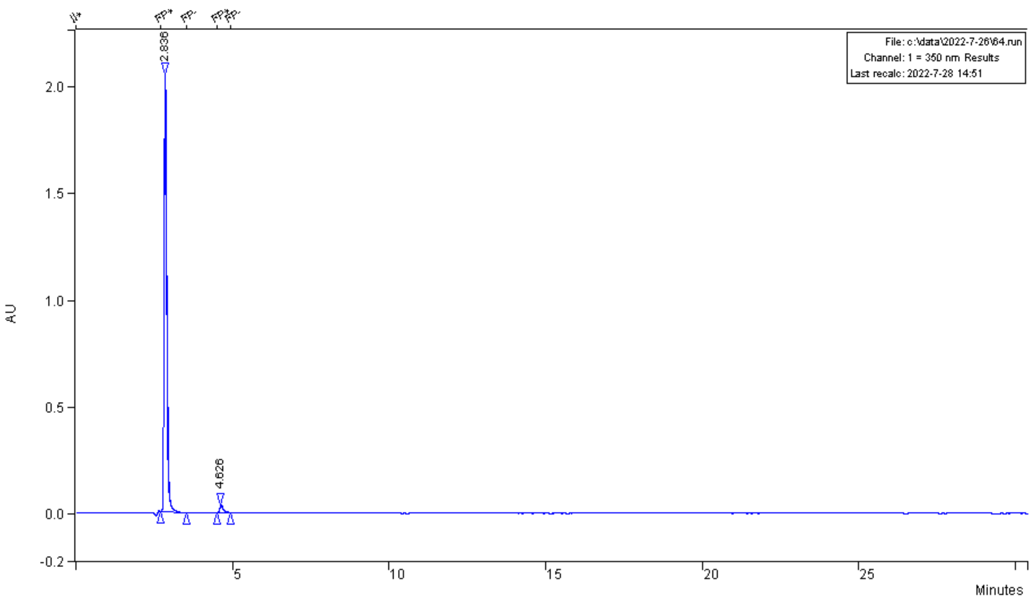

Figure S21. Purity test of compound **B5** by HPLC

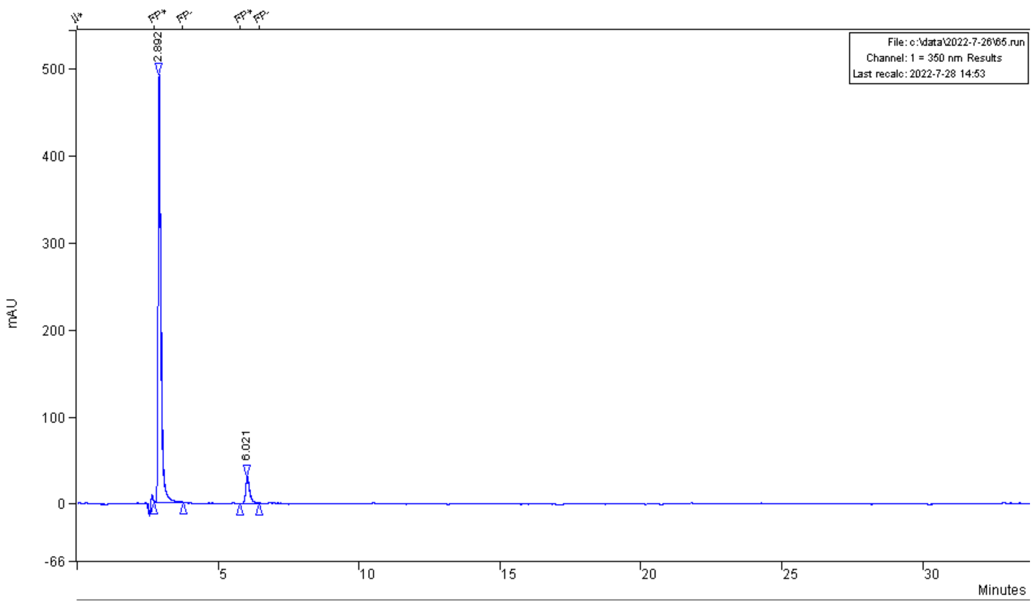

Figure S22. Purity test of compound **B6** by HPLC

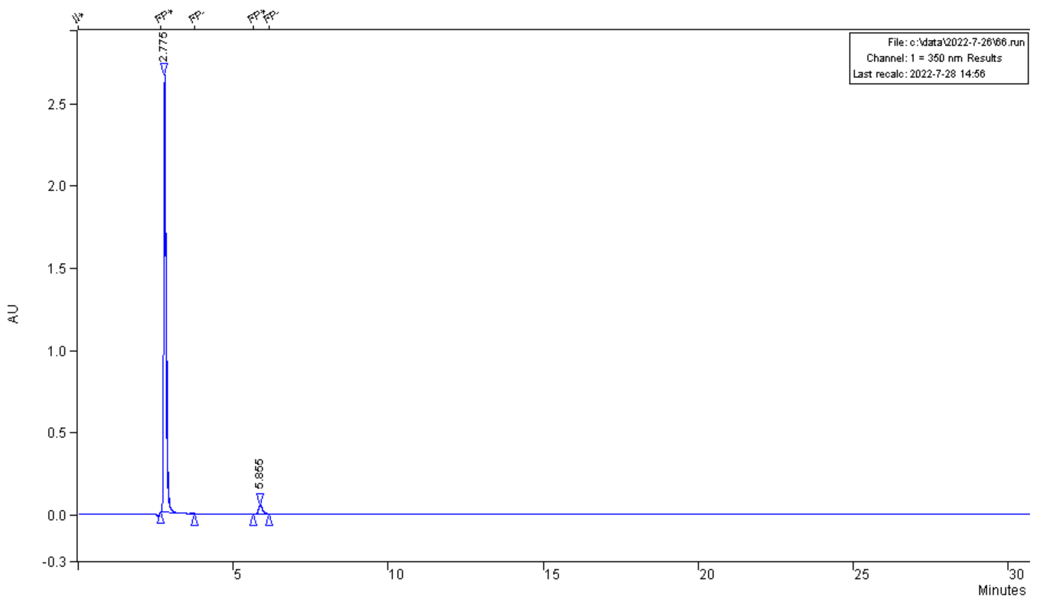

Figure S23. Purity test of compound **B7** by HPLC

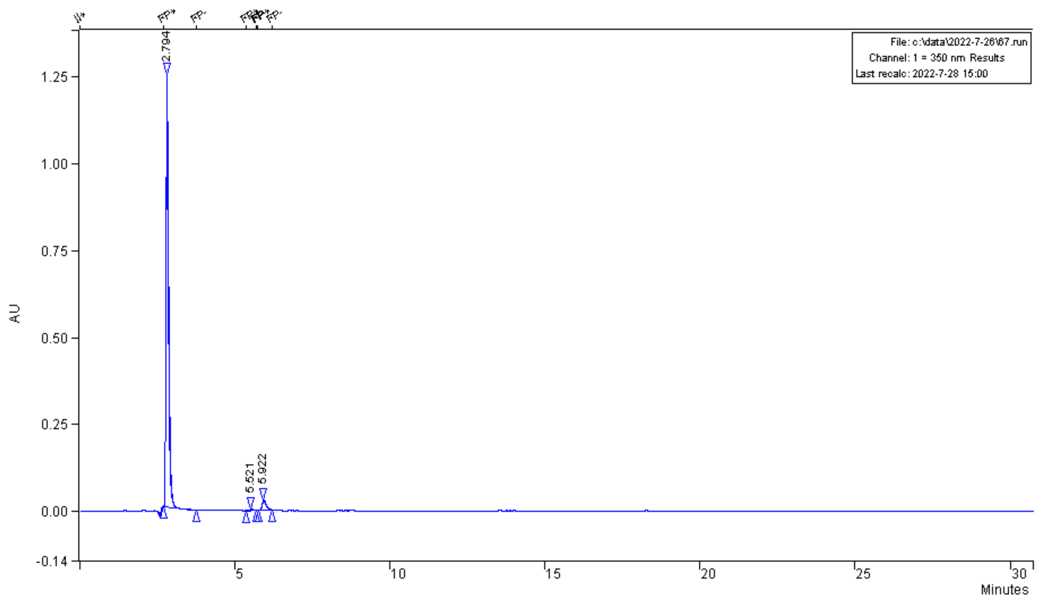

Figure S24. Purity test of compound **B8** by HPLC

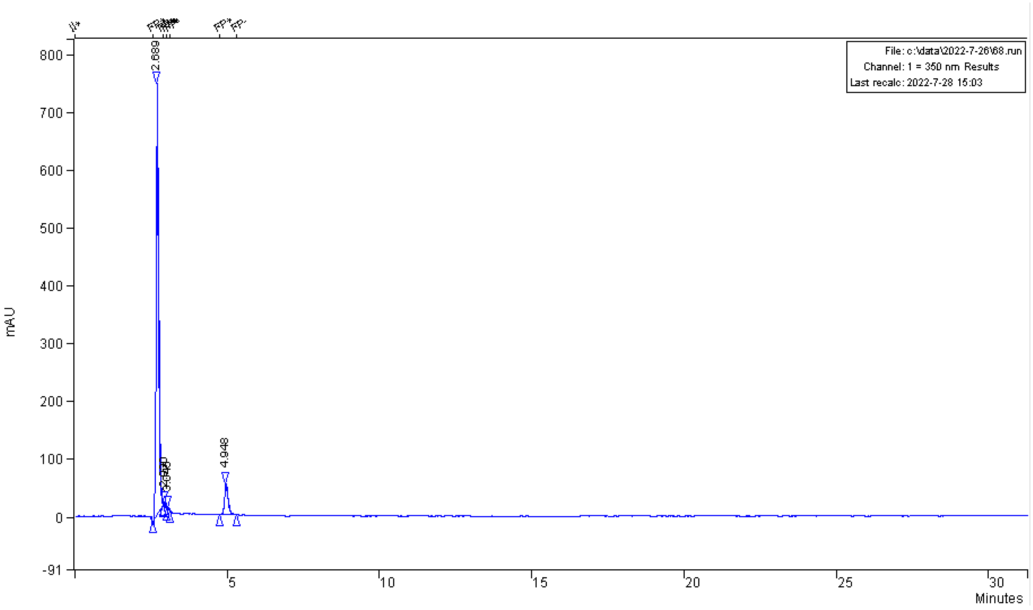

Figure S25. Purity test of compound **B9** by HPLC

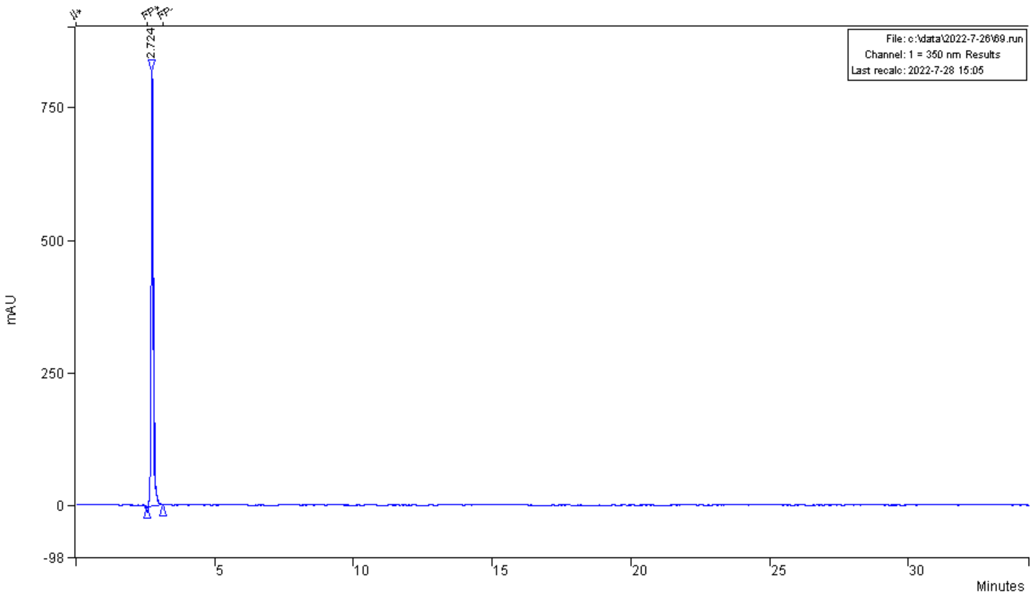

Figure S26. Purity test of compound **C1** by HPLC

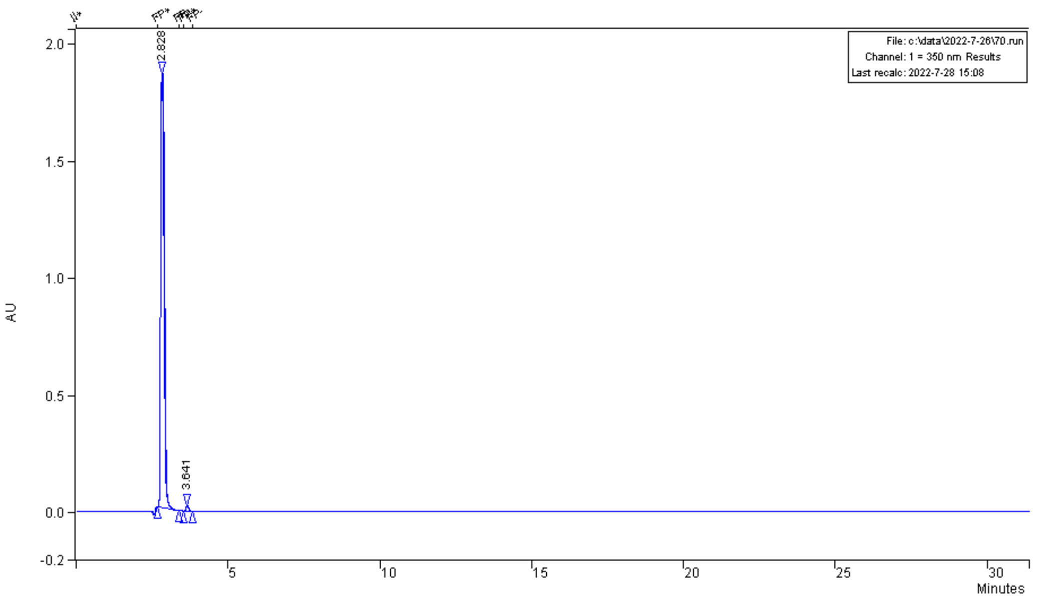

Figure S27. Purity test of compound **C2** by HPLC

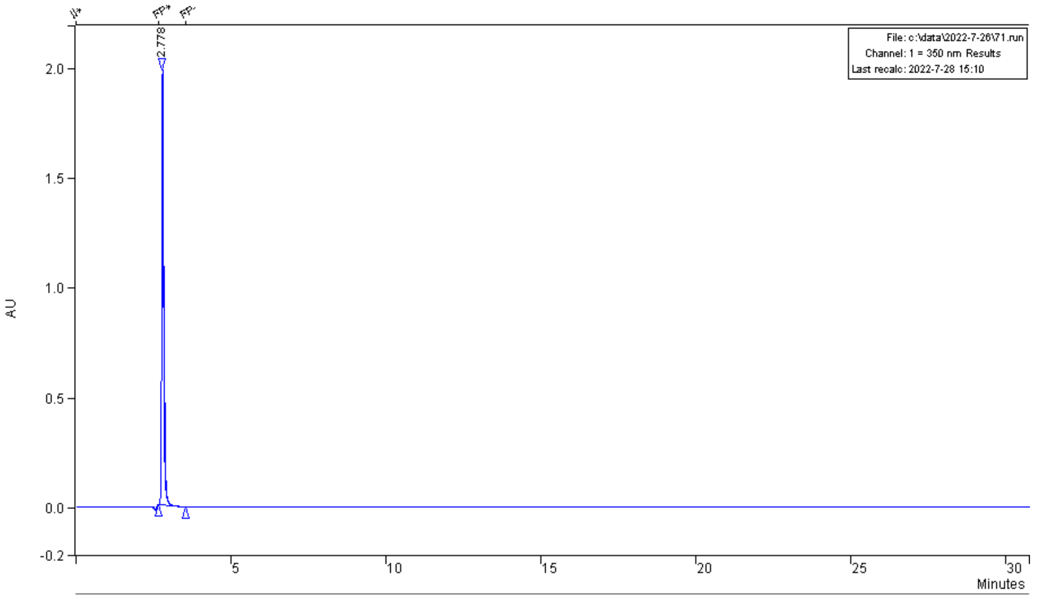

Figure S28. Purity test of compound **C3** by HPLC

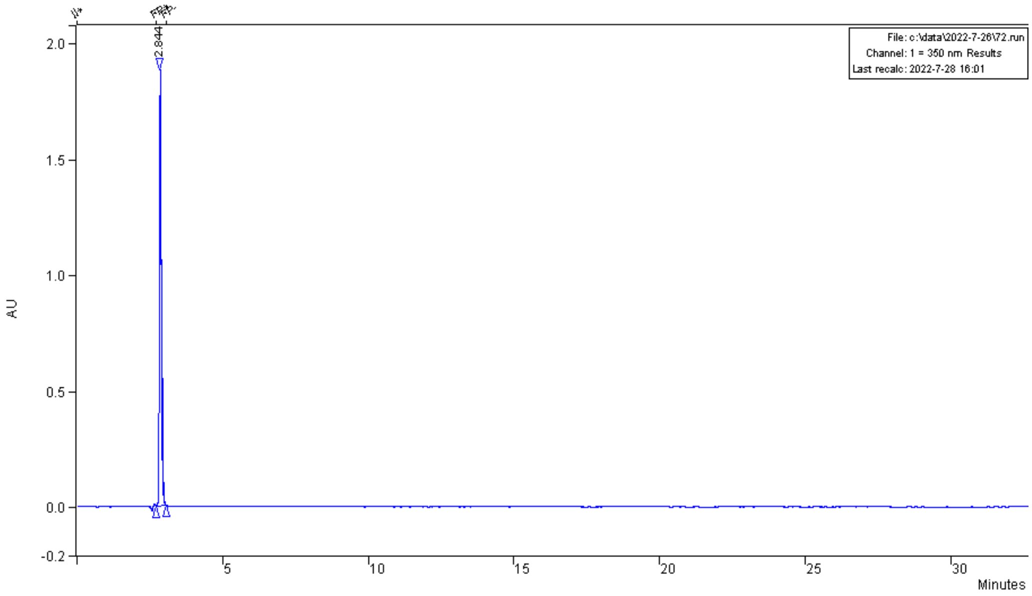

Figure S29. Purity test of compound **C4** by HPLC

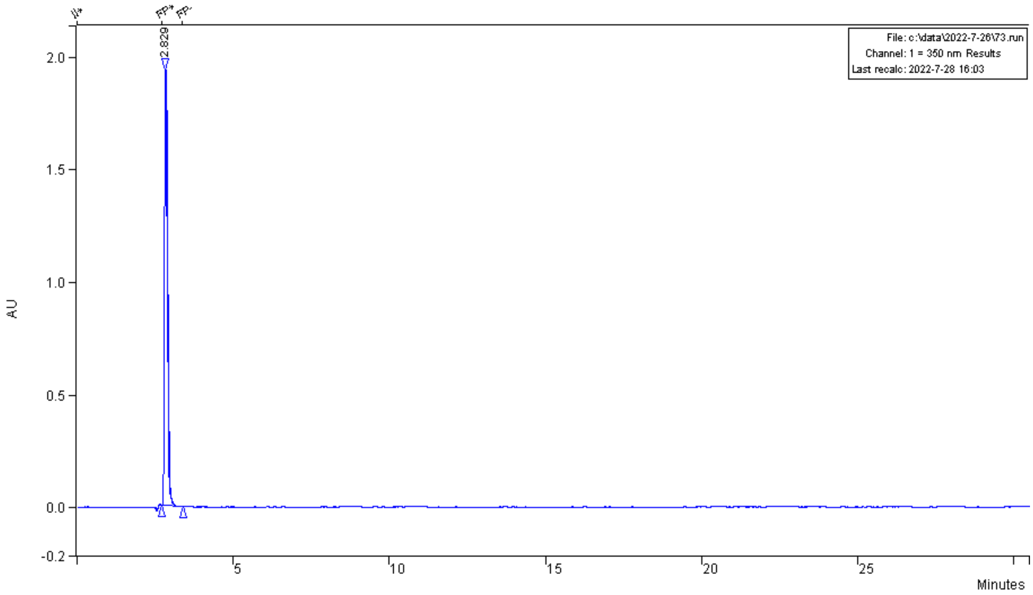

Figure S30. Purity test of compound **C5** by HPLC

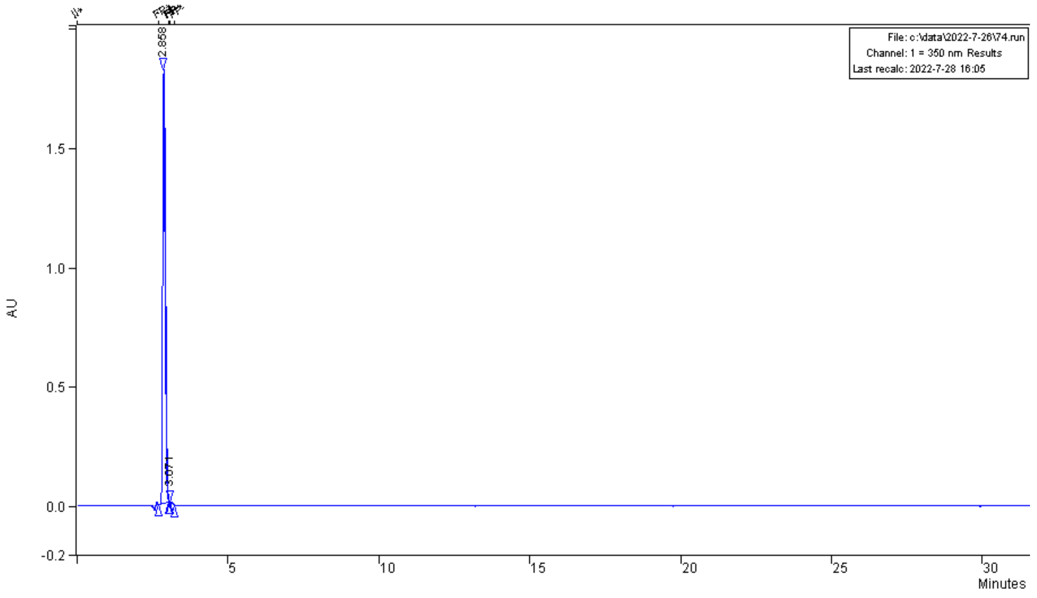

Figure S31. Purity test of compound **C6** by HPLC

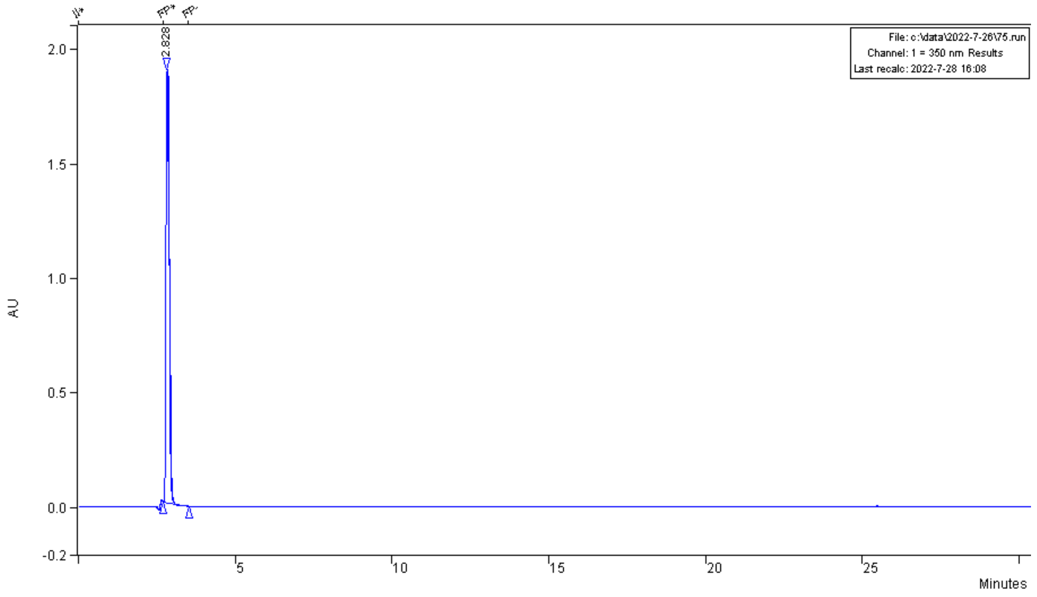

Figure S32. Purity test of compound **C7** by HPLC

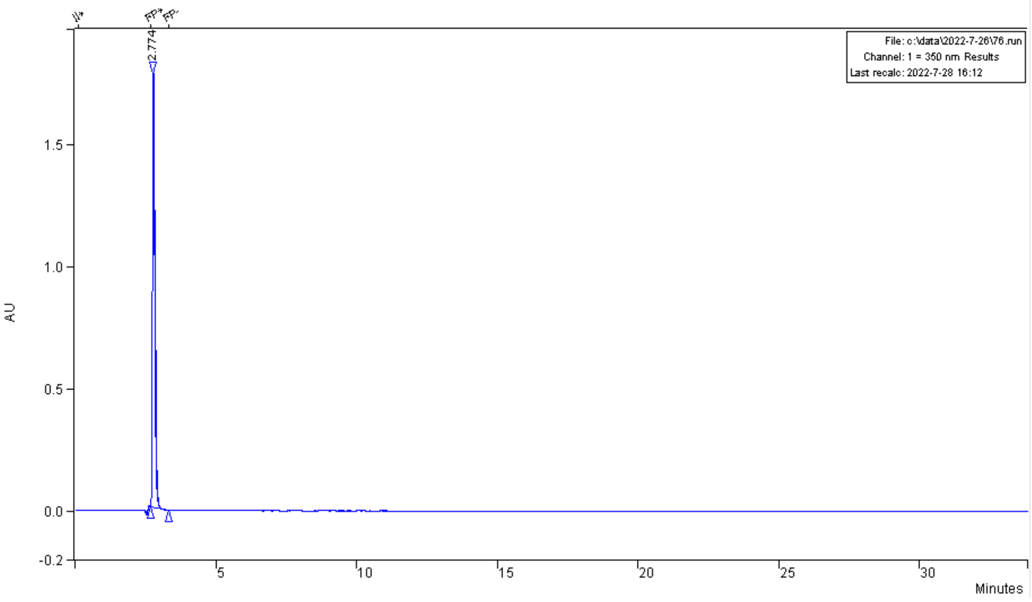

Figure S33. Purity test of compound **C8** by HPLC

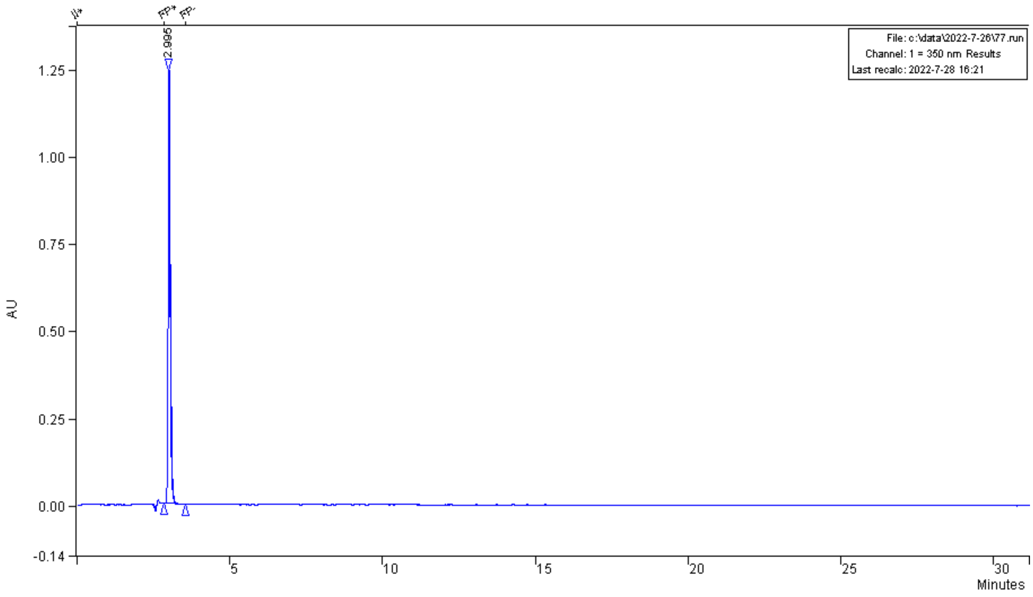

Figure S34. Purity test of compound **C9** by HPLC

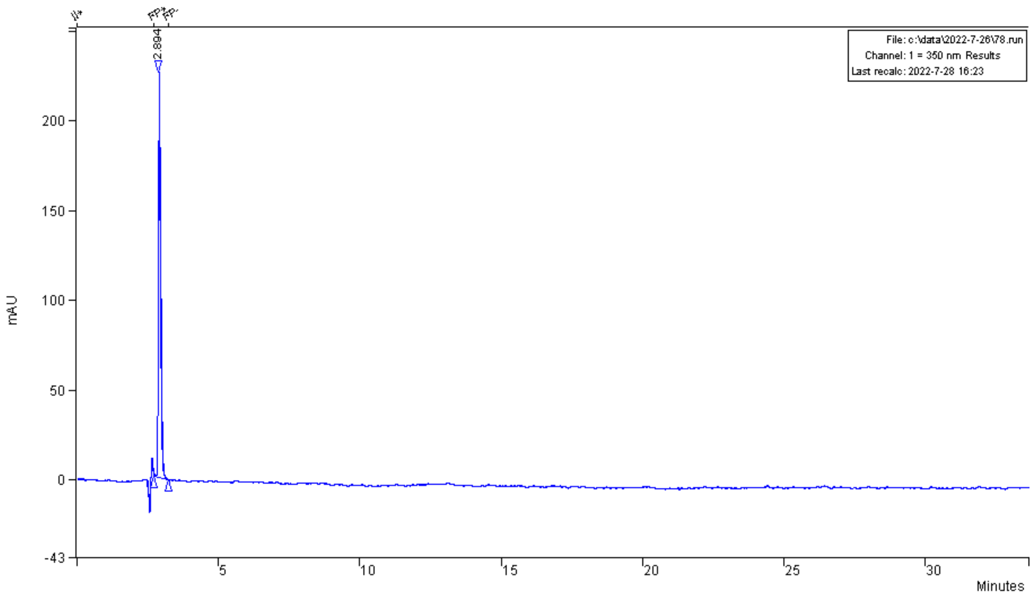

Figure S35. Purity test of compound **C10** by HPLC

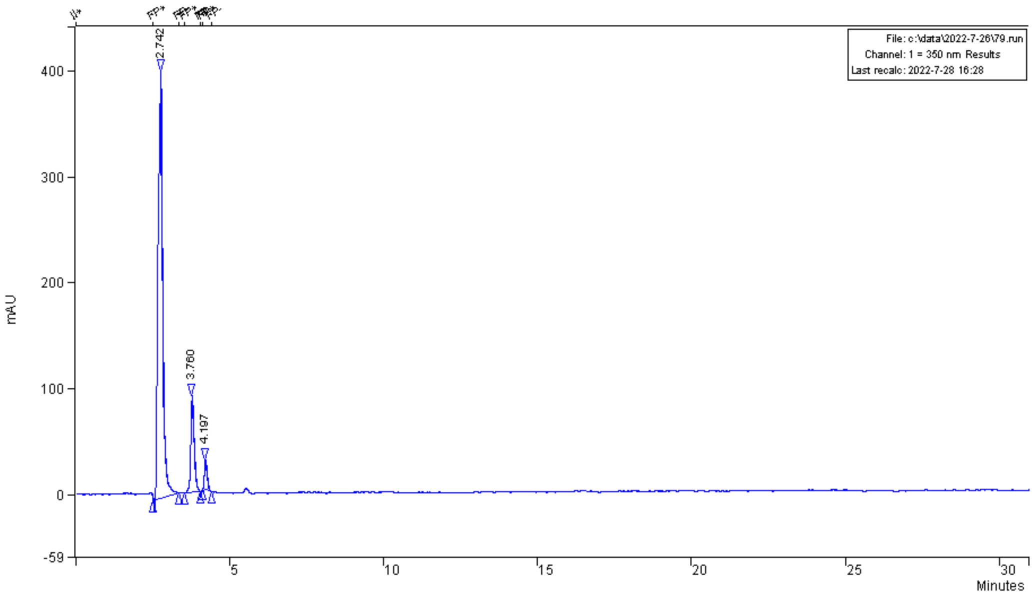

Figure S36. Purity test of compound **D1** by HPLC

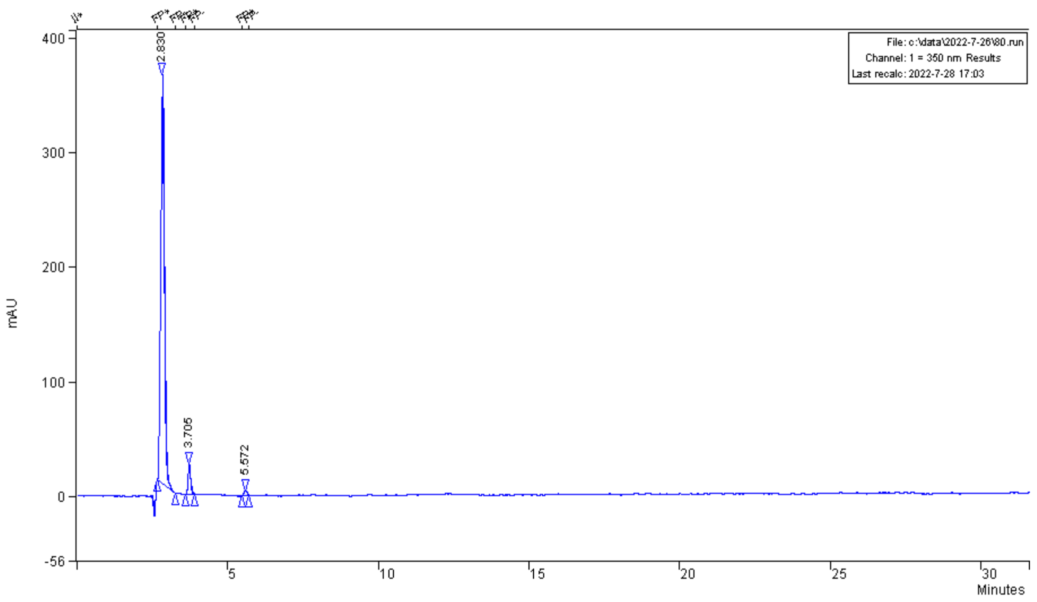

Figure S37. Purity test of compound **D2** by HPLC

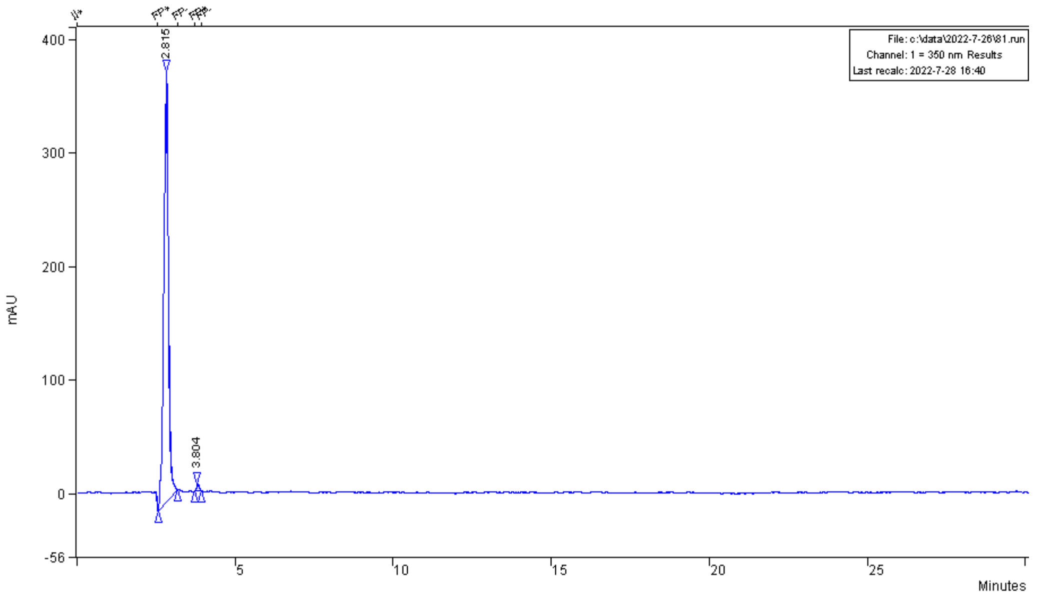

Figure S38. Purity test of compound **D3** by HPLC

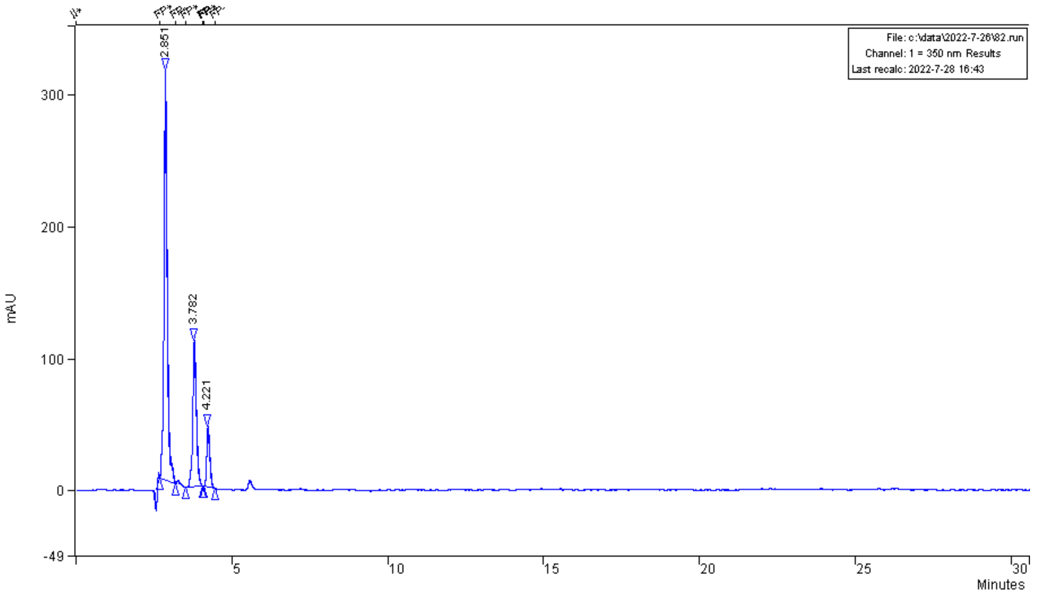

Figure S39. Purity test of compound **D4** by HPLC

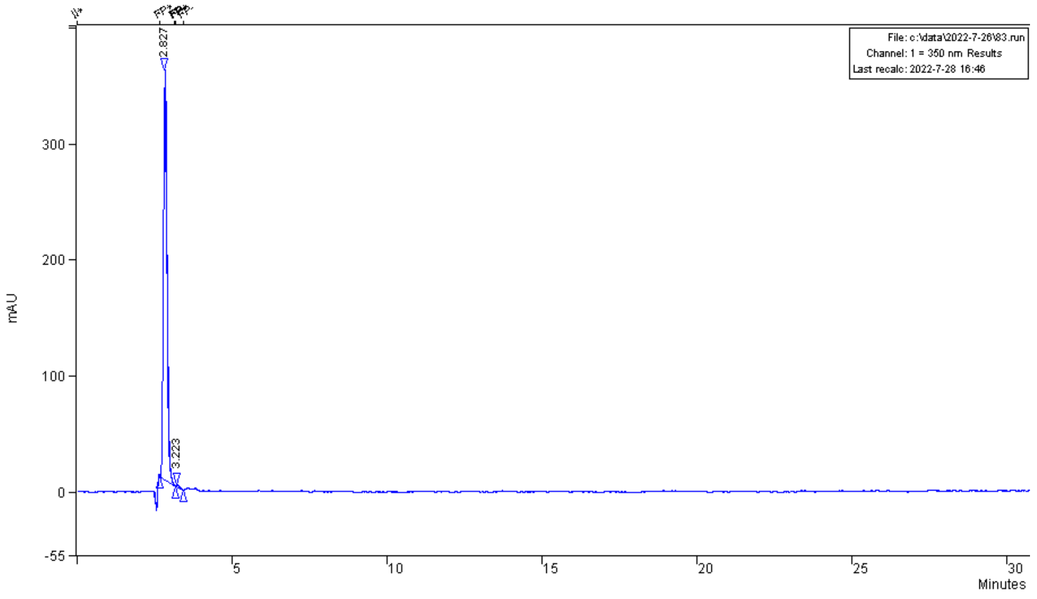

Figure S40. Purity test of compound **D5** by HPLC

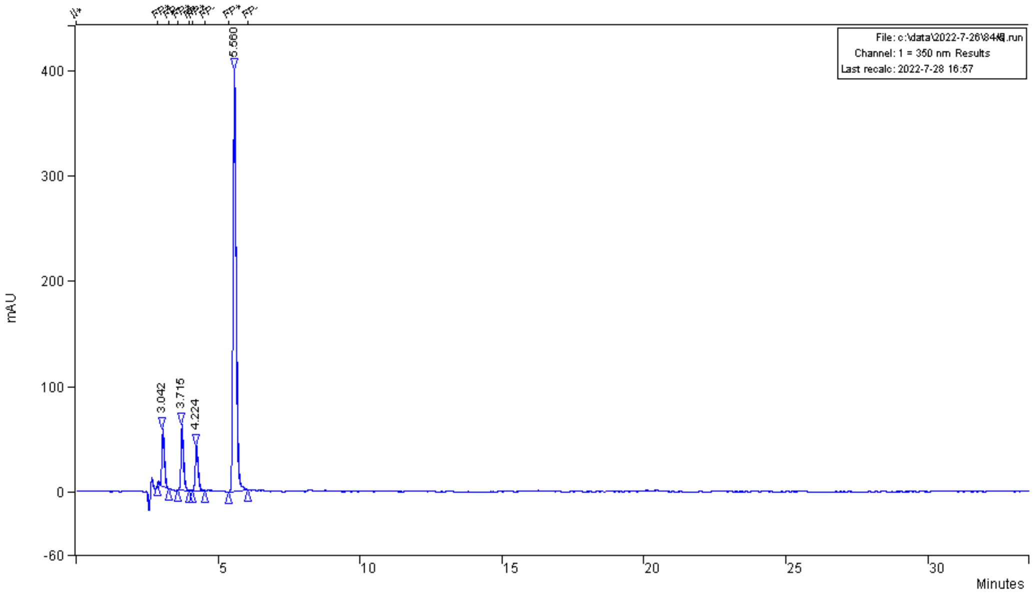

Figure S41. Purity test of compound **D6** by HPLC

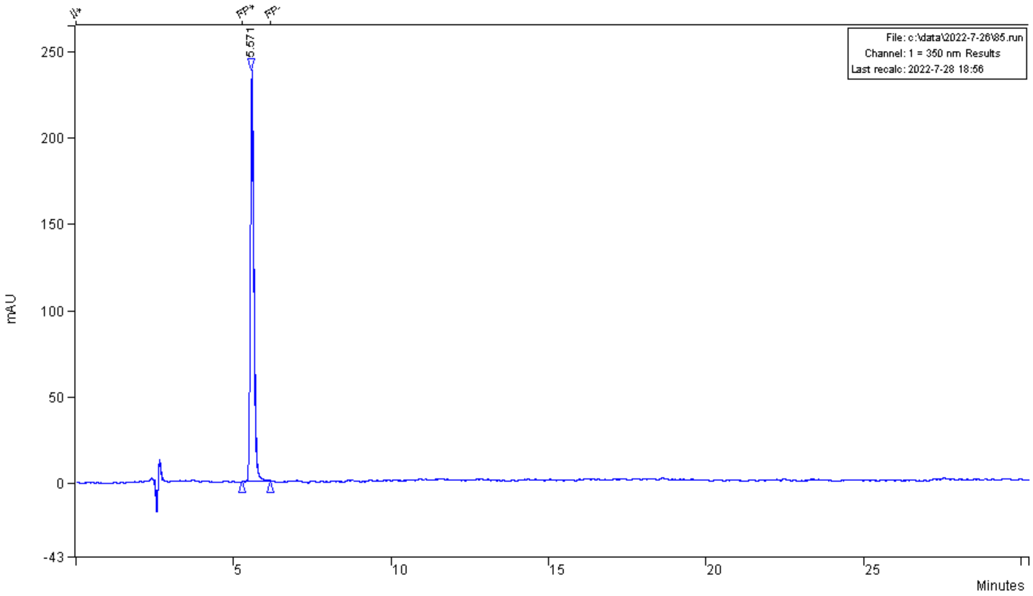

Figure S42. Purity test of compound **E1** by HPLC
